# Automatic calibration for on-line process monitoring in continuous-flow systems

**DOI:** 10.1155/S1463924695000022

**Published:** 1995

**Authors:** Manuel Agudo, Angel Ríos, Miguel Valcárcel, Andrei Danet

**Affiliations:** 1 Department of Analytical Ckemistry University of Córdoba Córdoba E-14004 Spain; 2 Faculty of Chemistry University of Bucharest Bul. Carol I Bucharest 13 Romania

## Abstract

An automatic procedure for calibration in continuous-flow systems is proposed. A flow gradient unit in the sample channel, controlled via a switching valve, delivers a concentration gradient of the standard so that the standard concentration varies with time after the flow-rate gradient is started. Thus, the signal (absorbance)- concentration relationship can be determined and checked for (recalibration) as frequently as required. The procedure was applied to a simulated continuous monitoring of hydrazine in waters, as well as to specific water samples that were spiked with hydrazine at different concentrations.


Journal of Automatic Chemistry, Vol. 17, No. 1 (January–February 1995), pp. 17–20

### Automatic calibration for on-line process monitoring in continuous-flow systems

**Manuel Agudo, Angel Ríos, Miguel Valcárcel**

*Department of Analytical Chemistry, University of Córdoba, E-14004 Córdoba, Spain*

**and Andrei Danet**
*Faculty of Chemistry, University of Bucharest, Bul. Carol I, 13, Bucharest, Rumania*

*An automatic procedure for calibration in continuous-flow systems is proposed. A flow gradient unit in the sample channel, controlled via a switching valve, delivers a concentration gradient of the standard so that the standard concentration varies with time after the flow-rate gradient is started. Thus, the signal (absorbance)-concentration relationship can be determined and checked for (recalibration) as frequently as required. The procedure was applied to a simulated continuous monitoring of hydrazine in waters, as well as to specific water samples that were spiked with hydrazine at different concentrations.*

### Introduction

Conventional routine work with continuous-flow systems involves manual preparation of the calibration solutions from a stock standard solution and subsequent sequential processing of diluted standard solutions, which means that sample analyses need to be stopped. Some calibration procedures can be integrated with the analyte determination in a flow manifold. Tyson and Appleton [1] used concentration gradients established by a dilution-calibration mini-apparatus for calibration in FI-atomic absorption spectrometry (the exponential calibration method). Another approach involves coupling a completely continuous system for the analyte determination with periodic injections of standards at different concentrations [2]. Automatic calibration and dilution in unsegmented flow systems can be also performed by including an open–closed calibration (dilution) loop for external standardization [3]. The following procedure was originally used to monitor residual chloride and nitrite in waters; it has recently been adapted for the standard-addition method for direct analysis of food samples for glucose and fructose [4].

The aim of this work was to develop a simple calibration unit for integration into a completely continuous-flow system intended for continuous monitoring of an analytical parameter in liquids. The object was to accomplish calibration and recalibration in a very short time. For this purpose, a flow-rate gradient unit [5] was incorporated into a flow manifold. The analytical potential of the flow-rate gradient technique has been demonstrated in a number of applications [6–8], and can be used for external calibration as it provides controlled, reproducible concentration gradients in the flow system. Such a unit was included in a manifold for continuous monitoring of hydrazine in waters.

### Experimental

### Reagents

All chemicals employed were of analytical-reagent grade. Distilled oxygen-free water was used to prepare all the solutions. 2,4-Dimethylaminobenzaldehyde reagent was prepared by dissolving 20 g of 2,4-dimethylaminobenzaldehyde (Merck) in 300 ml of methanol and 50 ml of concentrated hydrochloric acid and diluting to 500 ml with oxgyen-free water, after which it was stored in the dark.

Stock standard solution of hydrazine (Merck), 1·000 $gl^{-1}$ was also used.

### Apparatus

A Hewlett-Packard 8451A diode-array spectrophotometer, equipped with an HP 9121 floppy disk drive, an HP 7470A plotter and an HP 98155A keyboard was used. An Eppendorf switching valve, a Gilson Minipuls-3 peristaltic pump controlled by a Commodore-64 microcomputer through a laboratory-made interface, a Gilson Minipuls-2 peristaltic pump, a Hellma 178.12 QS flow-cell (inner volume 18 μl), and a Tecator TM III chemifold were also employed.

### Manifold

The manifold used is shown in figure 1. The analyte was monitored in a completely continuous flow system where the sample (or standard solution when required) was merged with a 0·5 M HCl stream ($R_1$ in figure 1) and then with the reagent stream (2,4-dimethylaminobenzaldehyde; $R_2$ in figure 1). Because the reaction was not very fast, a long reactor coil (R) was used before the photometric detector. The switching valve (SV) was used to introduce the sample and was actuated to perform calibrations at fixed times. Only water was introduced into channel 1 through SV (figure 1) at the beginning of the calibration procedure. The two pumps (PP = programmable pump; NPP = non programmable pump) were arranged to allow the flow-rate of channel 1 to be kept constant and the flow-gradient to be established via PP. To do this, the flow rate provided by NPP in channel 1 has to be higher than the maximum flow-rate yielded by PP. The auxiliary water stream continuously offset this flow-rate difference, thereby allowing a concentration gradient of standard to be generated in channel 1 with no change in the overall flow-rate.

### Results and discussion

Hydrazine converts 2,4-dimethylaminobenzaldehyde into its hydrazone, which absorbs at 460 nm [9]. Experimental

0142-0453/95 $10.00 © 1995 Taylor & Francis Ltd.

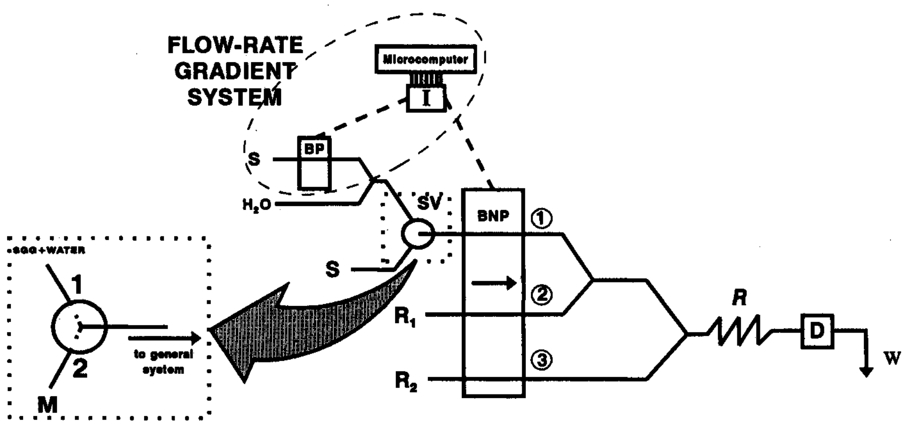

*Figure 1. Manifold used for automatic calibration with flow-rate gradients.*

variables involved in this reaction were initially optimized for increased sensitivity because the reaction is not very fast. The optimum concentrations for the chemicals involved were 4% (w/v) of reagent $R_2$ and 0·5 M HCl for $R_1$. The influence of the reactor coil length was studied between 50 and 1200 cm (0·8 mm, i.d.). A coil length of 625 cm was adopted. The flow-rates used were as follows: 1·4 ml/min for the sample/standard stream, and 0·4 ml/min for both reactant streams ($R_1$ and $R_2$).

### Characterization of concentration gradients

The principal advantage of the proposed procedure is that the calibration graph (equation) can be obtained by using a single standard solution. For this purpose, the concentration gradient generated by PP must be perfectly characterized. The high reliability of the system used to establish the gradients allows the signal–concentration relationship to be characterized accurately and precisely. The standard concentration at the detection point changes with time by virtue of the flow-gradient created by PP:

$$C_t = kC_0 q_t/q \qquad (1)$$

where $C_t$ and $C_0$ are the concentration at time t (min) and the initial concentration of the standard, respectively; $q_t$ and $q$ are the flow-rate at time t and the overall flow-rate, respectively; and $k$ is a proportionality constant. On the other hand, the flow-gradient, $Q$, is given by:

$$Q(\text{ml min}^{-2}) = dq/dt \qquad (2)$$

where

$$q_t = Qt$$

if $Q$ is constant (for example in the flow-gradients used in this work). Therefore, a combination of equations (1) and (2) gives:

$$C_t = kC_0 Qt/q. \qquad (3)$$

Because $k$, $Q$, $C_0$ and $q$ are constant under given experimental conditions, the relationship between $C_t$ and $t$ is as simple as:

$$C_t = kt \qquad (4)$$

or

$$C_t = k'C_0 t \qquad (5)$$

if more than one standard solution is used.

Thus, by establishing a flow-gradient $Q = 0{\cdot}0133$ ml min$^{-2}$ over an interval of 90 s, the following relationship was obtained:

$$C_t = 0{\cdot}0065\, C_0 t \quad \text{(time, in seconds)}.$$

Table 1 shows the times at which different concentrations of hydrazine were detected by using three different stock standard solutions. Any standard solution can be used to run the calibration plot because every signal corresponds to the same hydrazine concentration, irrespective of the stock standard solution employed. The most suitable $C_0$ value, however, will be a function of the hydrazine concentration level in the sample. Table 2 gives the calibration equations obtained by using three different

*Table 1. Concentration of hydrazine at the detection point determined with a single flow-gradient (0·0133 ml min$^{-2}$) and three different stock standard solutions ($C_0$).*

| Hydrazine concentration (μg/ml) | $t$ (s)* | | |
|---|---|---|---|
| | $C_0$ = 0·5 μg/ml | $C_0$ = 1·0 μg/ml | $C_0$ = 1·5 μg/ml |
| 0·05 | 15·4 | 7·7 | 5·1 |
| 0·10 | 30·8 | 15·4 | 10·2 |
| 0·14 | 43·1 | 21·5 | 14·3 |
| 0·25 | 77·0 | 38·5 | 25·6 |
| 0·40 | — | 61·5 | 41·0 |
| 0·57 | — | 87·7 | 58·5 |
| 0·70 | — | — | 71·8 |
| 0·85 | — | — | 87·2 |

*Time taken by the hydrazine concentration to reach the detection point.

*Table 2. Calibration equations obtained for three different stock standard solutions ($C_0$) by using a flow-gradient of 0·0133 ml min$^{-2}$.*

| $C_0$, μg/ml | Calibration equation* | Regression coefficient |
|---|---|---|
| 0·5 | $A$ = 1·066 [$N_2H_4$] + 0·004 | 0·9986 |
| 1·0 | $A$ = 1·103 [$N_2H_4$] + 0·008 | 0·9991 |
| 1·5 | $A$ = 1·169 [$N_2H_4$] + 0·016 | 0·9950 |

*$A$ = absorbance; hydrazine concentration in μg/ml.

stock standard solutions. The average relative standard deviation (r.s.d.) was ±1·5%.

*Analytical applications*

The performance of the proposed system was tested by using different synthetic samples of hydrazine. The results obtained are listed in table 3. A stock standard solution of 1·0 μg/ml of hydrazine was used and a calibration step included after each three samples (this unusually high calibration frequency is only justified for checking purposes). Figure 2 shows the recordings obtained. Table 4 lists the results obtained for hydrazine added to various real matrices (waters).

Some analytical process control applications entail continuous or near-continuous monitoring of the analyte. The proposed methodology can be very useful for this purpose as it allows a completely continuous-flow system to be assembled by incorporating the above described automatic calibration flow-gradient unit. Figure 3 shows the recordings obtained for simulated hydrazine control: three samples containing different concentrations of the analyte were monitored over 3 h. The figure also shows the most suitable calibration plot for each hydrazine level as automatically obtained by using hydrazine standard solutions of 0·5, 1·0 and 1·5 μg/ml as stock solutions in the flow-gradient unit.

*Table 3. Results obtained in the determination of hydrazine in synthetic samples with automatic calibration.*

| Concentration added (μg/ml) | Concentration found (μg/ml) | Concentration added (μg/ml) | Concentration found (μg/ml) |
|---|---|---|---|
| 0·22 | 0·21 | 0·80 | 0·79 |
| 0·70 | 0·70 | 0·60 | 0·64 |
| 0·20 | 0·23 | 0·93 | 0·91 |
| 0·25 | 0·26 | 0·72 | 0·73 |
| 0·60 | 0·63 | 0·30 | 0·29 |
| 0·56 | 0·55 | 0·44 | 0·45 |

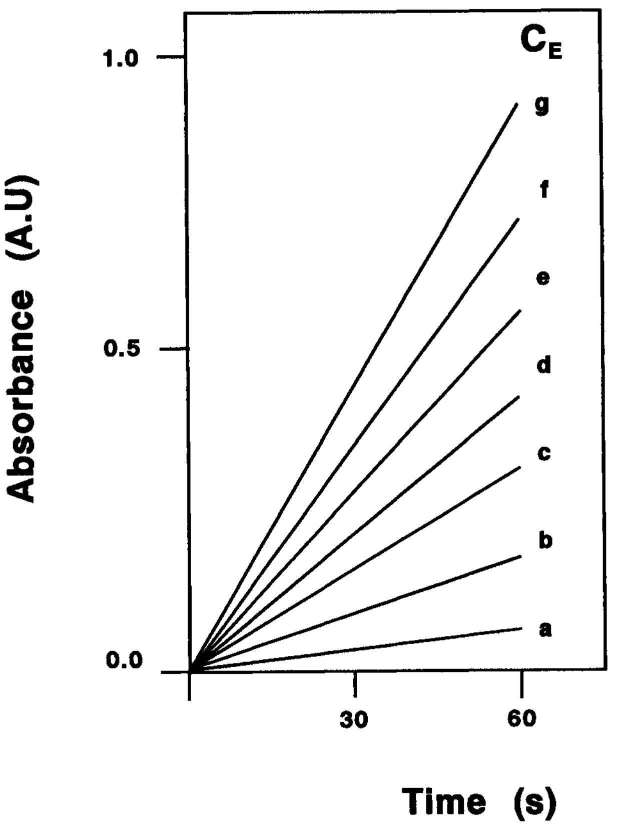

*Figure 2. Calibration graphs automatically obtained by the establishment of concentration gradients from different stock standard solutions: 0·2 μg/ml (a); 0·4 μg/ml (b); 0·6 μg/ml (c); 0·8 μg/ml (d); 1·2 μg/ml (e); 1.5 μg/ml (f); 1·8 μg/ml (g).*

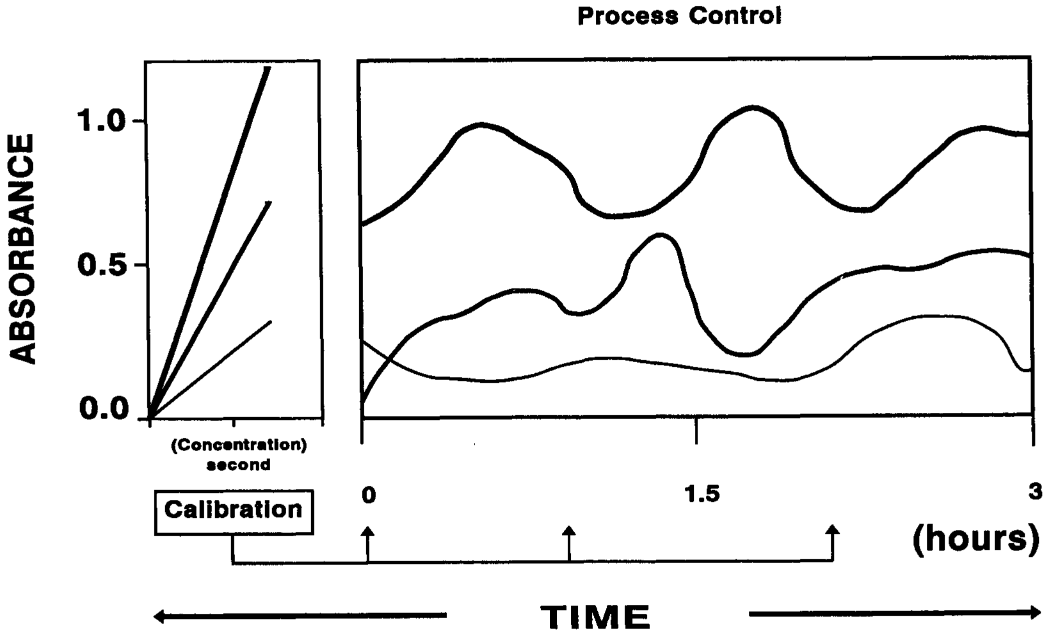

*Figure 3. Calibration graphs automatically recorded during a simulated hydrazine control experiment.*

*Table 4. Results obtained in the determination of hydrazine in real water matrices.*

| Type of water | Concentration added (μg/ml) | Concentration found (μg/ml) |
|---|---|---|
| Tap water (Villa del Río) | 0·60 | 0·66 |
| Tap water (Córdoba) | 0·60 | 0·64 |
| Well water | 0·50 | 0·58 |
| Lake water | 0·50 | 0·53 |
| River water (Guadalquivir) | 0·60 | 0·65 |
| Mineral water | 0·50 | 0·55 |

### Conclusion

The proposed flow-gradient unit for automatic calibration in continuous-flow systems is especially simple and flexible for incorporation into any flow manifold. When calibration is required, the sample stream is replaced with a short plug of standard that is delivered via a concentration gradient. As the calibration plug passes through, the detector allows the signal-concentration relationship to be readily determined for subsequently running a conventional calibration graph. Only a single stock standard solution is needed. This automatic calibration unit should be particularly useful in process control, whether periodic or continuous monitoring of an analyte is required.

### References

1. Tyson, J. F. and Appleton, J. M. H., *Analytical Proceedings*, **22** (1985), 17.
2. Tyson, J. F. and Idris, A. B., *Analyst*, **109** (1984), 23.
3. Agudo, M., Ríos, A. and Valcárcel, M., *Analytica Chimica Acta*, **264** (1993), 265.
4. Agudo, M. Ríos, A. and Valcárcel, M., *Flow Analysis VI* (Toledo, Spain, 1994).
5. Agudo, M., Marcos, J., Ríos, A. and Valcárcel, M., *Analytica Chimica Acta*, **239** (1990). 211.
6. Marcos, J., Ríos, A. and Valcárcel, M., *Trends in Analytical Chemistry* (1992).
7. Lendl, B., Grassenbauer, M., Ríos, A. and Valcárcel, M., *Analytica Chimica Acta*, **289** (1994), 187.
8. Marcos, J. Ríos, A. and Valcárcel, M., *Flow Analysis VI* (Toledo, Spain, 1994).
9. Basson, W. D. and Van Staden, J. F., *Analyst*, **103** (1978), 998.

